# Evaluating minimal important differences and responder definitions for the asthma symptom diary in patients with moderate to severe asthma

**DOI:** 10.1186/s41687-019-0109-2

**Published:** 2019-04-03

**Authors:** Gary Globe, Ingela Wiklund, Maria Mattera, Hao Zhang, Dennis A. Revicki

**Affiliations:** 1 Amgen, Thousand Oaks, CA USA; 2 Gothenburg University, Gothenburg, Sweden; 3 Patient Reported Outcome Consortium, Critical Path Institute, Tucson, AZ USA; 4 Patient-Centered Research, Evidera, Bethesda, MD USA

**Keywords:** Asthma, Asthma symptom diary, Interpretation guidelines, Minimal important difference, Responder definitions, Responsiveness to change

## Abstract

**Background:**

The Asthma Symptom Diary was developed to assess severity of symptoms in patients with moderate to severe asthma, and has evidence supporting reliability and validity. Only limited information is available on sensitivity to change and responder definitions for the Asthma Symptom Diary.

**Objectives:**

Main study objectives were to evaluate sensitivity to change and provide responder definitions for clinically meaningful effects for the Asthma Symptom Diary.

**Methods:**

This is a secondary analysis of Phase II clinical trial data in patients with moderate to severe asthma, Asthma Symptom Diary (ASD) was collected daily during the 24-week study. The Asthma Control Questionnaire and the Patient Global Assessment were collected at baseline, and week 12 and 24. Analysis of covariance (ANCOVA) models were used to evaluate sensitivity to change in Asthma Symptom Diary scores after 12 and 24 weeks of treatment. Anchor-based methods, using Asthma Control Questionnaire and Patient Global Assessment defined anchors, were used to identify minimal important differences and various responder criteria for changes in mean 7-day ASD score, symptomatic days, and minimal symptom days.

**Results:**

Sample was 59% female, 81% White, with a mean age of 47.3 (SD = 13.6) years. ANCOVAs demonstrated significant differences in baseline to week 12 and week 24 changes in mean 7-day Asthma Symptom Diary scores and symptomatic days by Asthma Control Questionnaire (all *p* < 0.001) and Patient Global Assessment anchors (all *p* < 0.001). Meaningful responders, from the patient’s perspective, were defined as improvements of 0.5–0.6 points (SD = 0.6; scale range 0 to 4) in mean 7-day Asthma Symptom Diary scores, and as a reduction of 2 to 3 Asthma Symptom Diary-based symptomatic days.

**Conclusion:**

The Asthma Symptom Diary was responsive to changes in clinical status in patients with moderate to severe asthma. Responder definitions were identified, including symptomatic days, for evaluating individual level treatment effects in clinical trials.

## Background

Asthma is a chronic respiratory illness characterized by airway remodeling, inflammation, and immunological hyper-responsiveness to allergens [22]. Approximately 300 million people worldwide suffer from asthma and many of these patients are poorly controlled [9]. Based on the 2014 Centers for Disease Control estimates, the overall prevalence of asthma is 8% in the US, with prevalence rates of 9% in children and 7% in adults [5]. In 2009, 52% of asthmatics (both adults and children) experienced an asthma attack that increased their risk for an emergency department visit or an inpatient hospitalization [1]. Patient reported symptoms and health-related quality of life (HRQL) are important measures of disease experience and impact of asthma, and previous research has demonstrated the HRQL burden of asthma [2, 6, 28–30].

Daily diaries have frequently been used to assess symptom severity and treatment effectiveness in asthma clinical trials [16, 20, 26]. However, few of these symptom diaries have been systematically developed, and have evidence supporting their reliability and validity, and established responder definitions [3, 25, 31]. The Food and Drug Administration (FDA) has recommended that patient reported outcome (PRO) measures, including daily symptom diaries, are developed based on patient qualitative information and that these measures have evidence supporting reliability, validity and responsiveness [8].

The Asthma Symptom Diary (ASD) was developed to assess severity of asthma symptoms, nocturnal awakenings and activity limitations in patients with moderate to severe asthma. The symptom content of the ASD was based on concept elicitation and cognitive interviews in patients with moderate to severe persistent asthma [10]. Previous studies have supported the content validity of the ASD [10] and the psychometric characteristics of the ASD [11]. The ASD has good evidence supporting reliability, validity and responsiveness of the average 7-day score. However, the original psychometric evaluation study was conducted in a sample of asthma patients in a 4-week observational study, limiting the evaluation of minimal important differences (MID) and providing no responder definitions, reflecting important changes from the patient’s perspective. The psychometric evaluation of a new measure, such as the ASD, is an ongoing exercise with additional evidence on reliability, validity, and interpretation guidelines providing additional confidence in the instrument’s measurement properties. The current psychometric analysis was designed to further confirm the measurement qualities of the ASD using data from a much larger, 24-week clinical trial.

Information on the interpretation and responder definitions of new PRO measures are needed to assist clinicians, patients and regulatory agencies in understanding the effectiveness of treatments. For regulatory agencies, such as the FDA and [7]). Evaluation of MID and responder definitions are critical for interpreting the effects of treatment on PRO measures, such as the ASD [4, 12, 23, 24]. The MID is often used for interpreting mean group differences between treatments and reflects the smallest score or change in scores on the PRO measure that would likely be important from the patient’s perspective [12, 23]. However, some researchers base responder definitions on the MID.

Responder definitions focus on the individual level and assist in understanding percent of patients benefiting from treatment. One definition of a responder is a patient who has experienced a change that is important to that patient, that is, has experienced the MID. Thus, the change as great or greater than the MID can be selected as the criterion for defining a responder, that is, the MID represents the threshold for response, though investigators may choose other thresholds (e.g. moderate or large responses). Responder definitions are based on a threshold of changes in endpoint scores based on psychometric evidence and are defined as a magnitude of change that is considered important to the patient. The patient’s perspective is critical, and meaningful change (or improvement) needs to be determined based on patient input. Responder definitions for interpreting individual patient-level change are normally larger than the MID (for group or individual level interpretation) [13, 19]. The US FDA has expressed concern that the MID, as a basis for defining responders, reflects minimal rather than more than minimal levels of change in PRO endpoints.

Both MIDs and responder definitions are based on anchor-based criteria measures [23]. These anchor scores may be patient derived (i.e., patient global assessment of change or severity), clinician derived (i.e., clinician global rating of change or severity), based on relevant clinical indicators (i.e., hematocrit), or other established PRO endpoints (i.e., Asthma Control Questionnaire; ACQ).

Responder definitions may differ between chronic symptomatic versus episodic symptomatic diseases. In chronic diseases (e.g., gastroparesis, cancer, congestive heart failure, etc.), responder definitions can be based on baseline to endpoint changes in scores, as these represent meaningful outcomes. For episodic diseases, where there are variations in symptomatic episodes, with relatively milder symptoms between episodes (e.g., asthma, celiac disease, migraine, etc.), a combination of clinical and psychometric criteria is most often used to determine responder definitions. Asthma, especially moderate to severe disease, has characteristics of both chronic and episodic conditions, with acute exacerbations occurring within this chronic condition, but less severe symptoms occurring between these exacerbations. Thus, in the current study, we evaluate changes in mean 7-day ASD scores and in symptomatic and minimal symptom days as potential endpoints for clinical trials comparing asthma treatments. Psychometric criteria are based on the anchor-based and distribution-based analyses, while clinical criteria are related to ensuring that any responder definition reflects the underlying clinical context, that is, represents meaningful information from the perspective of both the patient and the clinician.

In the current study, Phase II clinical trial data were analyzed to evaluate the MID and to identify various responder definitions based on the ASD measure in patients with persistent moderate to severe asthma. The main objective was to provide meaningful interpretation guidelines for the ASD with respect to its application in randomized clinical trials comparing asthma treatments.

## Methods

### Study design and patients

This was a secondary analysis of data collected from a Phase II clinical trial in patients with moderate to severe persistent asthma. The Phase II clinical trial was a randomized, double-blind, placebo-controlled, multiple dose study to evaluate the safety and efficacy of brodalumab in subjects with moderate to severe asthma (NCT01902290). The clinical trial involved 566 subjects with asthma recruited from 147 study centers worldwide. Study subjects were randomized in a 1:1 ratio to brodalumab 210 mg or placebo every two weeks with approximately 283 subjects per treatment group. This study enrolled men and women between 18 and 75 years of age, with a diagnosis of asthma, with inadequately controlled asthma (ACQ ≥ 1.5 at both screening and baseline), and with a forced expiratory volume in one second (FEV1) ≥ 40% and ≤ 80% (at screening and baseline). All subjects needed to be treated with a stable dose of ICS (≥ 200 and ≤ 1000/μg/day fluticasone powder or equivalent) and if on a long acting beta-adrenoceptor agonist, must have been on a stable dose. Additionally, subjects must have had ≥1, but < 5 exacerbations in the year before screening. Patients were excluded if they had an acute asthma exacerbation requiring emergency room treatment with systemic corticosteroids or hospitalization within 30 days of screening or any exacerbation between screening and baseline, use of systemic corticosteroids within the period starting 4 weeks before screening, ≥ 5 asthma exacerbations in the year prior to screening, history of chronic obstructive pulmonary disease or other chronic pulmonary condition, or sleep apnea, respiratory infection, any clinically significant and unstable systemic disease, or pregnant or breastfeeding.

Following randomization, subjects received brodalumab 210 mg or placebo. The primary endpoint was change in asthma control (based on the ACQ; [17]) from baseline to week 24, and all subjects were followed for up to 24 weeks. The clinical trial protocol was approved by an Institutional Review Board (Chesapeake Research Review; Pro00007797), and each patient provided written informed consent before participating in the study. This psychometric analysis was based on pooled and masked PRO and other clinical data from the treatment and placebo groups in the Phase II clinical trial.

### Measures

#### Asthma symptom diary

The ASD was developed to evaluate the severity of asthma-related symptoms, nocturnal awakenings and activity limitations based on a daily diary which is completed in the morning and evening each day [10, 11]. The ASD contains 10 questions, with 5 questions completed every morning and another 5 questions completed every evening. The morning diary comprises questions on four asthma-related symptoms (wheezing, shortness of breath, cough, chest tightness), rated using a 5-point severity scale from 0 (no symptom) to 4 (very severe symptoms), and one question on nocturnal awakenings, rated on a scale from 0 (did not wake up) to 4 (unable to sleep due to asthma). The evening diary has questions on the same four asthma-related symptoms and one question on limitations of activities, rated on a scale from 0 (not at all) to 4 (extremely). The ASD daily score is computed by averaging the responses to the 10 items, and a mean 7-day ASD score is calculated by averaging the 7 daily scores (range 0 to 4). The daily score is not calculated if any item responses are missing. The mean ASD 7-day score is only calculated if at least 4 of 7 ASD daily scores are not missing. The ASD has evidence supporting reliability (internal consistency, test-retest reliability), concurrent and known groups validity, and responsiveness [11]. The ASD was collected daily over the course of the clinical trial.

In addition to the average 7-day ASD scores, three other scores were derived from the ASD daily scores based on previous ASD data [11] and clinician review. Definitions of Symptomatic Days and Minimum Symptom Days were based on daily ASD scores, and Symptomatic and Minimal Symptom Days were examined over 7-day time periods. In addition, mean changes in average 7-day ASD scores were also evaluated.

The ASD-based endpoints were: (1) Symptomatic Days (defined as mean of the 10 ASD daily symptom items ≥1, otherwise non-Symptomatic Day); (2) Minimal Symptom Days-1 (defined as mean of the 10 ASD daily symptom items ≤1 and no single symptom item score > 1, otherwise non-Minimal Symptom Day-1); and (3) Minimal Symptom Days-2 (defined as no single ASD daily symptom item score > 1, and activity limitations or nighttime awakening item scores = 0, otherwise non-Minimal Symptom Day-2).

#### Asthma control questionnaire

The ACQ is a 7-item disease-specific instrument designed to assess asthma control [17, 18]. Five items assess asthma-related symptoms and activity limitations; one item on FEV1% predicted; and one item on beta-agonist use. All seven items are scored on a 7-point scale, with 0 indicating good control and 6 indicating poor control; the total score is the mean of the seven items. A change of 0.50 points is considered clinically meaningful [18] and a total score of < 1.0 indicates good asthma control [15, 21, 27]. Analyses were completed using the 5-item ACQ total score (which excludes the FEV1 and beta-agonist questions). ACQ-5 was collected at baseline and at every study visit; the baseline, week 12 and week 24 data was used in this analysis.

#### Patient global assessment (PGA)

The PGA was a single-item patient-rated assessment of disease severity on a scale of 0 (no asthma symptoms) to 5 (very severe). The PGA measures the patient’s asthma disease state at the time of assessment and was measured at every study visit. The PGA response scale was 0 = no asthma, 1 = very mild, 2 = mild, 3 = moderate, 4 = severe, and 5 = very severe.

#### Statistical analysis

This secondary statistical analysis was based on those patients with complete baseline to 12-week and baseline to 24-week ASD data. All the psychometric data analyses were conducted masked to treatment group, as the focus on this study was the measurement performance of the ASD. No adjustments were made for multiplicity and the nominal *p*-values < 0.05 were used to evaluate statistical significance.

### Descriptive statistics

Demographic and clinical characteristics for the study sample were summarized using descriptive statistics (mean, standard deviation, and range for quantitative variables; frequency, and percentage for categorical variables).

### Responsiveness (sensitivity to change)

Ability to detect change is a type of validity in health outcomes measurement and refers to the extent to which the instrument can detect change in the predicted direction when there has been a notable change in patient status [14, 23]. Anchor-based methods are recommended for evaluating MID, and for identifying responder definitions for PRO measures. Distribution-based methods may be helpful in supporting the anchor-based findings. We first examined the correlations between baseline to 12 and 24 weeks changes in ASD scores and changes in the ACQ and PGA scores using Spearman correlations to ensure that the anchors were sufficiently correlated with the ASD [23].

Analysis of covariance (ANCOVA) was used to examine the difference in the mean change scores of average 7-Day ASD scores from baseline to weeks 12 and 24 between ACQ and PGA defined responder and non-responder groups, controlling for age, gender, FEV1% predicted, and baseline mean 7-Day ASD score. A significant main effect of the overall model at *p* < 0.05, and a significant post-hoc difference among each pairwise comparison (p < 0.05) was considered supportive of the responsiveness of the ASD scores. Effect size was also estimated for the ACQ and PGA defined responder and non-responder groups.

Ability of the ASD to detect change was assessed by comparing changes in mean 7-Day Asthma Symptom Diary scores. ASD change scores were evaluated from baseline to weeks 12 and 24 based on the ACQ-5 and PGA responder groups. For the responsiveness analyses, responders and non-responders were defined as follows:

- ACQ-5 score: responders were defined as patients with an ACQ-5 change score of ≤ − 0.5 from baseline to weeks 12 and 24; and non-responders were defined as patients with an increase in ACQ-5 score or a decrease in ACQ-5 score of less than 0.5 from baseline to weeks 12 and 24. Previous research has identified a change of 0.5 in ACQ scores as meaningful for patients [18].
- ACQ-5 score: responders were defined as patients with an ACQ-5 change score ≤ − 1.0 from baseline to weeks 12 and 24; and non-responders were defined as patients with an increase in ACQ-5 score or a decrease in ACQ-5 score of less than 1.0 from baseline to weeks 12 and 24. This anchor responder definition was used as a more stringent indicator of treatment response based on twice the ACQ MID.
- PGA score: responders were defined as patients with a ≥ 1 or more change from baseline to weeks 12 or 24; and non-responders were defined as patients with a < 1 change from baseline to weeks 12 and 24. For the PGA, a one-unit change is the smallest possible improvement (or worsening) that can be observed. The one-unit improvement in PGA scores may be considered the MID.

### Evaluation of MID and responder definitions

ANCOVAs were used to evaluate differences in mean 7-day ASD scores, and ASD-based 7-Day Symptomatic Days and Minimal Symptom Days responder definitions for change from baseline to weeks 12 and 24 as the dependent variables and change groups (based on ACQ) as independent variables, controlling for age, gender, FEV1% predicted, and baseline average 7-Day ASD score. Separate ANCOVA models were conducted for each responder definition based on the ACQ independent variables. A significant main effect of the overall model at *p* < 0.05, and a significant post-hoc difference among each pairwise comparison (p < 0.05) was considered supportive of the ACQ-based responder definition. The ACQ 0.5 and ACQ 1.0 anchors can be considered reflective of the MID and individual responder definitions, respectively.

In addition, cumulative distribution function (CDF) analysis was conducted based on the three anchor scales and changes in mean 7-day ASD scores, symptomatic days and minimal symptom days [4]. For CDFs, the continuous plot of change from baseline is included on the horizontal axis and the cumulative percentage of patients reporting up to that change is included on the vertical axis.

## Results

### Sample characteristics

These analyses are based on the 417 (74%) of study participants with complete baseline and 12-week follow-up ASD data, and complete baseline to 24-week follow-up ASD data (*n* = 345, 61%). The total sample was 59% female, 81% White, with a mean age of 47.3 (SD = 13.6) years. Mean number of years with asthma was 22.6 (SD = 14.6, range 0.4 to 62 years). For the overall analytic sample, mean baseline 7-day ASD scores were 0.97 (SD = 0.6), and mean baseline ACQ scores were 2.5 (SD = 0.8). Mean baseline Symptomatic Days scores were 3.33 (SD = 3.01), Minimal Symptom Days-1 scores were 3.01 (SD = 2.85), and Minimal Symptom Days-2 scores were 1.43 (SD = 2.27).

### Correlations between changes in ASD scores and changes in ACQ and PGA scores

The Spearman correlations between baseline to 12-week changes in ASD scores and baseline to 12-week changes in ACQ and PGA scores were 0.59 and 0.57, respectively. The correlations between baseline to 24-week changes in ASD scores and baseline to 24-week changes in ACQ and PGA scores were 0.67 and 0.53, respectively.

### Responsiveness (sensitivity to change)

#### Mean 7-day ASD scores

The results of the ANCOVA evaluating differences in mean 7-day ASD scores by the ACQ and PGA anchors are summarized in Table 1. At week 12, statistically significant differences were observed in mean 7-day ASD scores based on the ACQ 0.5 (*p* < 0.001) and ACQ 1.0 (*p* < 0.001) definitions of responders. For example, based on the ACQ 0.5 responder criteria, responders reported a mean change of − 0.49 points compared to − 0.05 points for non-responders on the average 7-day ASD (difference 0.43 points; *p* < 0.001). The baseline to week 12 changes in average 7-day ASD scores were similar for the PGA based responder groups (− 0.48 versus -0.07; difference 0.41 points; p < 0.001). Comparable, although somewhat larger differences between ACQ or PGA based responders and non-responders, were seen at week 24 (p < 0.001; Table 1). Effect sizes for the anchor-based responder groups ranged from 0.80 to 1.13 for mean 7-day ASD scores.

**Table 1:** Responsiveness of the Average 7-Day ASD Score at Weeks 12 and 24

	Responders Mean (SE)	Non-Responders Mean (SE)	Difference	P-Value
Week 12				
ACQ > 0.5	−0.49 (0.03)	− 0.05 (0.03)	− 0.43	*p* < 0.001
Effect size	0.82	0.08		
ACQ > 1.0	−0.54 (0.03)	−0.13 (0.03)	− 0.42	*p* < 0.001
Effect size	0.90	0.22		
PGA	−0.48 (0.03)	−0.07 (0.03)	− 0.41	*p* < 0.001
Effect size	0.80	0.12		
Week 24				
ACQ > 0.5	−0.59 (0.03)	−0.06 (0.03)	− 0.53	*p* < 0.001
Effect size	0.98	0.10		
ACQ > 1.0	−0.68 (0.04)	−0.15 (0.03)	− 0.53	*p* < 0.001
Effect size	1.13	0.25		
PGA	−0.60 (0.03)	−0.10 (0.04)	− 0.49	*p* < 0.001
Effect size	1.00	0.17		

#### ASD-based symptomatic days

The ANCOVA results evaluating differences in ASD based Symptomatic Days by the ACQ and PGA anchors are summarized in Table 2. At week 12, statistically significant differences were observed in Symptomatic Days based on the ACQ 0.5 responder criteria (*p* < 0.001) and the ACQ 1.0 responder criteria (*p* < 0.001). For example, based on the ACQ 0.5 responder criteria, responders reported a mean decrease of − 2.21 Symptomatic Days compared with − 0.57 days for non-responders (difference 1.64 days; *p* < 0.001). The baseline to week 12 changes in Symptomatic Days were similar for the PGA based responder groups (− 2.34 versus − 0.45; difference 1.86 days; p < 0.001). Comparable, although larger differences between ACQ or PGA based responders and non-responders, were seen in Symptomatic Days in the week 24 analyses (all p < 0.001; Table 2). Effect sizes for the anchor-based responder groups ranged from 0.73 to 1.07 for Symptomatic Days.

**Table 2:** Responsiveness of ASD Symptomatic Days in a 7-Day Period at Weeks 12 and 24

	Responders Mean (SE)	Non-Responders Mean (SE)	Difference	*P*-Value
Week 12				
ACQ > 0.5	−2.21 (0.16)	−0.57 (0.18)	−1.64	*p* < 0.001
Effect size	0.73	0.19		
ACQ > 1.0	−2.35 (0.20)	−0.90 (0.16)	−1.45	*p* < 0.001
Effect size	0.78	0.30		
PGA	−2.34 (0.16)	−0.45 (0.17)	−1.86	*p* < 0.001
Effect size	0.78	0.15		
Week 24				
ACQ > 0.5	−2.86 (0.18)	− 0.28 (0.28)	− 2.57	*p* < 0.001
Effect size	0.95	0.09		
ACQ > 1.0	−3.21 (0.21)	−0.77 (0.20)	−2.44	*p* < 0.001
Effect size	1.07	0.26		
PGA	−2.97 (0.19)	−0.45 (0.23)	−2.52	*p* < 0.001
Effect size	0.99	0.15		

Symptomatic Days defined as mean of the 10 ASD daily symptom items ≥1, otherwise non-Symptomatic Day

#### ASD-based minimal symptom days

For the first definition of Minimal Symptom Days (i.e., mean of the 10 ASD daily symptom items ≤1 and no single symptom item score > 1), the ANCOVA results evaluating differences in ASD based Minimal Symptom Days by ACQ and PGA-based responders are summarized in Table 3. At week 12, statistically significant differences were observed in Minimal Symptom Days-1 for the ACQ 0.5 responder criteria (*p* < 0.001) and ACQ 1.0 responder criteria (p < 0.001). Based on the ACQ 0.5 responder criteria, responders reported a mean increase of 2.29 Minimal Symptom Days-1 compared with 0.61 days for non-responders (difference 1.68 days; *p* < 0.001). The baseline to week 12 changes in Minimal Symptom Days-1 were similar for the PGA based responder groups (2.46 versus 0.48; difference 1.98 days; *p* < 0.001). Comparable, although larger differences between ACQ or PGA based responders and non-responders, were seen in changes in mean Minimal Symptom Days-1 in the week 24 analyses (all *p* < 0.001; Table 3).

**Table 3:** Responsiveness of ASD Minimal Symptom Days in a 7-Day Period at Weeks 12 and 24

	Responders Mean (SE)	Non-Responders Mean (SE)	Difference	P-Value
Minimal Symptom Days-1				
Week 12				
ACQ > 0.5	2.29 (0.17)	0.61 (0.18)	1.68	*p* < 0.001
Effect size	0.80	0.21		
ACQ > 1.0	2.41 (0.20)	0.96 (0.16)	1.45	*p* < 0.001
Effect size	0.85	0.34		
PGA	2.46 (0.17)	0.48 (0.17)	1.98	*p* < 0.001
Effect size	0.86	0.17		
Week 24				
ACQ > 0.5	2.91 (0.18)	0.50 (0.24)	2.41	*p* < 0.001
Effect size	1.02	0.18		
ACQ > 1.0	3.21 (0.21)	0.98 (0.20)	2.23	*p* < 0.001
Effect size	1.13	0.34		
PGA	3.01 (0.19)	0.63 (0.23)	2.38	*p* < 0.001
Effect size	1.06	0.22		
Minimal Symptom Days-2				
Week 12				
ACQ > 0.5	1.93 (0.16)	0.04 (0.17)	1.97	*p* < 0.001
Effect size	0.85	0.02		
ACQ > 1.0	2.29 (0.19)	0.29 (0.15)	2.05	*p* < 0.001
Effect size	1.01	0.13		
PGA	1.79 (0.17)	0.20 (0.18)	1.59	*p* < 0.001
Effect size	0.79	0.09		
Week 24				
ACQ > 0.5	2.26 (0.18)	0.06 (0.28)	2.20	*p* < 0.001
Effect size	1.00	0.03		
ACQ > 1.0	2.61 (0.21)	0.44 (0.19)	2.17	*p* < 0.001
Effect size	1.15	0.19		
PGA	2.14 (0.19)	0.42 (0.24)	1.72	*p* < 0.001
Effect size	0.94	0.19		

Minimal Symptom Days-1 defined as mean of the 10 ASD daily symptom items ≤1 and no single symptom item score > 1

Minimal Symptom Days-2 defined as no single ASD daily symptom item score > 1, and activity limitations or nighttime awakening item scores = 0

For the second definition of Minimal Symptom Days (i.e., no single ASD daily symptom item score > 1, and activity limitations or nighttime awakening item scores = 0), the findings of the ANCOVA evaluating differences in ASD based Minimal Symptom Days by ACQ and PGA-based responders are summarized in Table 3. At week 12, statistically significant differences were observed in Minimal Symptom Days-2 for the ACQ 0.5 responder criteria (*p* < 0.001) and ACQ 1.0 criteria (*p* < 0.001). Based on the ACQ 0.5 responder criteria, responders reported a mean increase of 1.93 Minimal Symptom Days-2 compared to a decrease of 0.04 days for non-responders (difference 1.97 days; *p* < 0.001). The baseline to week 12 changes in Minimal Symptom Days-2 were similar for the PGA based responder groups (1.79 versus 0.20; difference 1.59 days; *p* < 0.001). Comparable, although larger differences between ACQ or PGA based responders and non-responders, were seen in changes in mean Minimal Symptom Days-2 in the baseline to week 24 analyses (all *p* < 0.001; Table 3). Effect sizes for anchor-based responder groups range from 0.80 to 1.13 for Minimal Symptoms Days-1 and 0.79 to 1.15 for Minimal Symptom Days-2.

### MID and responder definitions

#### Mean 7-day ASD scores

The results of the ANCOVA evaluating differences in mean 7-day average ASD scores by the ACQ anchors are summarized in Table 4. The ACQ 0.5 and ACQ 1.0 anchors can be considered reflective of the MID and individual responder definitions, respectively. At week 12, statistically significant differences were observed in mean 7-day ASD scores based on the ACQ change groups (*p* < 0.0001). For example, the ACQ 0.5 change group reported a mean change of − 0.35 and the ACQ 1.0 change group reported a mean change of − 0.54 points on the average 7-day ASD scores compared with a change on only − 0.05 points for the ACQ > 0.5 group (differences all *p* < 0.01). Comparable differences between ACQ based change groups were observed in average 7-day ASD scores in the week 24 analyses (all *p* < 0.01; Table 4). Therefore, the MID for mean 7-day ASD scores may be 0.35.

**Table 4:** Changes in Average 7-Day ASD Score by ACQ Change Groups at Weeks 12 and 24

ACQ Group	Week 12	Week 24
1 Change ≤ −1.0 [Responder]	−0.54	−0.68
2 Change −0.5 to −1.0 [MID]	−0.35	−0.35
3 Change ≥ −0.5	− 0.05	− 0.05
Model p-value	< 0.0001	< 0.0001
Significant contrasts (p < 0.01)	1v2; 1v3; 2v3	1v2; 1v3; 2v3

#### ASD-based symptomatic days

The results of the ANCOVA evaluating differences in mean 7-day average ASD scores by the ACQ anchors are summarized in Table 5. At week 12, statistically significant differences were observed in changes in ASD Symptomatic Days based on the ACQ change groups (*p* < 0.0001). For example, the ACQ 0.5 change group reported a mean change of − 1.75 (i.e., MID) and the ACQ 1.0 change group reported a mean change of − 2.34 points in ASD Symptomatic Days compared with a change of only − 0.61 days for the ACQ > 0.5 group (differences all *p* < 0.01). Comparable, although somewhat larger, differences between ACQ based change groups were seen in changes in ASD Symptomatic Days in the week 24 analyses (all *p* < 0.01; Table 5).

**Table 5:** Changes in ASD Symptomatic Days in a 7-Day Period by ACQ Change Groups at Weeks 12 and 24

ACQ Group	Week 12	Week 24
1 Change ≤ −1.0 [Responder]	−2.34	−3.22
2 Change −0.5 to −1.0 [MID]	− 1.75	−1.98
3 Change ≥ − 0.5	− 0.61	−0.35
Model *p*-value	< 0.0001	< 0.0001
Significant contrasts (p < 0.01)	1v3; 2v3	1v2; 1v3; 2v3

#### ASD-based minimal symptom days

For the first definition of Minimal Symptom days (i.e., mean of the 10 ASD daily symptom items ≤1 and no single symptom item score > 1), the ANCOVA results evaluating differences in Minimal Symptom Days by ACQ-based change groups are summarized in Table 6. At week 12, statistically significant differences were observed in Minimal Symptom Days-1 for the ACQ 0.5 change group (*p* < 0.01) and ACQ 1.0 change group (*p* < 0.01), compared to the ACQ > 0.5 change group. The ACQ 0.5 change group reported a mean increase of 1.97 Minimal Symptom Days-1 compared with an increase of 0.6 days for no change group (difference 1.37 days; *p* < 0.01). Comparable, although larger, differences between ACQ based change groups were seen in changes in mean Minimal Symptom Days-1 in the week 24 analyses (all *p* < 0.01; Table 6).

**Table 6:** Changes in ASD Minimal Symptom Days in a 7-Day Period by ACQ Change Groups at Weeks 12 and 24

ACQ Group	Minimal Symptom Days-1		Minimum Symptom Days-2	
	Week 12	Week 24	Week 12	Week 24
1 Change ≤ −1.0 [Responder]	2.43	3.23	2.31	2.56
2 Change −0.5 to −1.0 [MID]	1.97	2.16	1.02	1.36
3 Change ≥ −0.5	0.60	0.54	−0.01	0.08
Model p-value	< 0.0001	< 0.0001	< 0.0001	< 0.0001
Significant contrasts (p < 0.01)	1v3; 2v3	1v2; 1v3; 2v3	1v3; 2v3	1v2; 1v3; 2v3

Minimal Symptom Days-1 defined as mean of the 10 ASD daily symptom items ≤1 and no single symptom item score > 1

Minimal Symptom Days-2 defined as no single ASD daily symptom item score > 1, and activity limitations or nighttime awakening item scores = 0

For the second definition of Minimal Symptom Days (i.e., no single ASD daily symptom item score > 1, and activity limitations or nighttime awakening item scores = 0), the findings of the ANCOVA evaluating changes in ASD based Minimal Symptom Days by ACQ-based responders are summarized in Table 6.

At week 12, statistically significant differences were observed in Minimal Symptom Days-2 for the ACQ 0.5 responder criteria (*p* < 0.01) and ACQ 1.0 change groups (*p* < 0.01). Those patients in the ACQ 0.5 change group reported a mean increase of 1.02 Minimal Symptom Days-2 compared to a decrease of 0.01 days for those in the ACQ > 0.5 change group (difference 1.01 days; *p* < 0.01). Comparable, although slightly larger, differences between ACQ change groups were seen in changes in Minimal Symptom Days-2 in the baseline to week 24 analyses (all *p* < 0.01; Table 6).

### Cumulative distribution functions

**Fig. 1:**
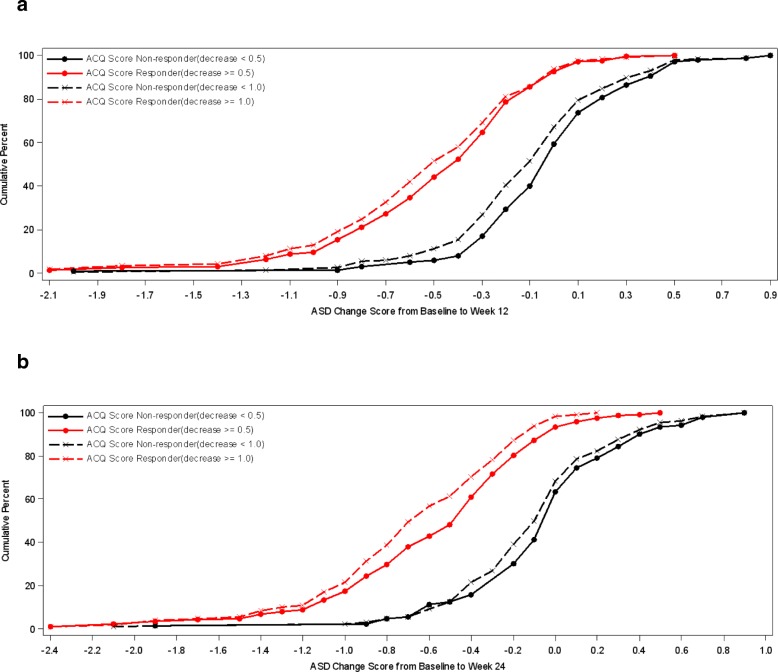
Cumulative Distribution Curves for 7-Day Average ASD Scores by ACQ Based Responder Criteria. **a** Baseline to Week 12 by ACQ-5 Response. **b** Baseline to Week 24 by ACQ-5 Response. PRO analysis set includes all subjects who are enrolled and have Asthma Symptom Diary measurement at baseline and at 12 weeks or 24 weeks

**Fig. 2:**
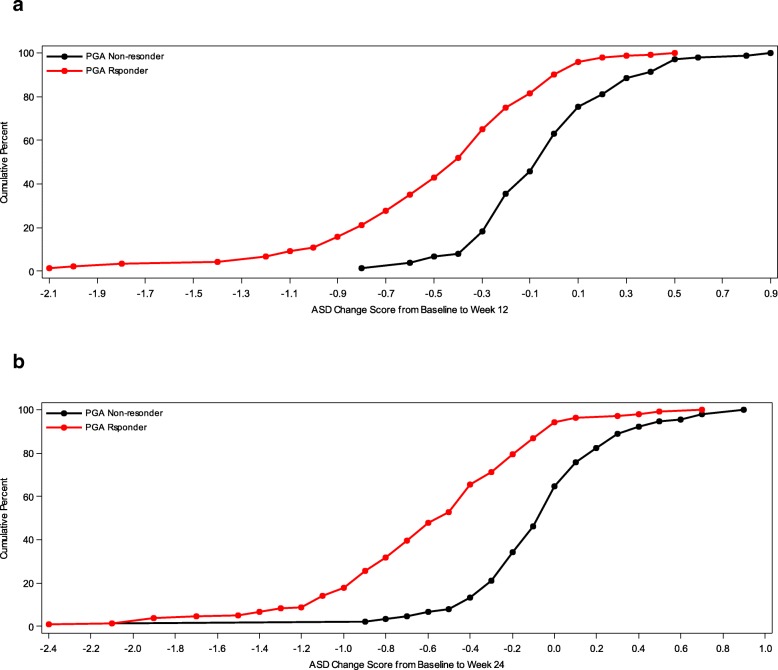
Cumulative Distribution Curves for 7-Day Average ASD Scores by PGA Based Responder Criteria. **a** Baseline to Week 12 by PGA Response. **b** Baseline to Week 24 by PGA Response. PRO analysis set includes all subjects who are enrolled and have Asthma Symptom Diary measurement at baseline and at 12 weeks or 24 weeks

Cumulative distribution function (CDF) analyses were conducted for 7-Day Average ASD scores, Symptomatic Days, and the two definitions of Minimal Symptom Days based on ACQ criteria of ≥0.5 improvements and based on PGA improvements of 1 or more at weeks 12 and 24. For 7-Day Average ASD scores, CDF results are displayed for the ACQ criteria and for the PGA criteria (see Figs 1 and 2). Clearly, there is discrimination in ASD scores across most of the distribution of changes for baseline to week 12 and baseline to week 24. Based on the CDF results, the responders demonstrated a decrease of 0.40 to 0.50 points in 7-Day Average ASD scores, while non-responders showed only a zero to 0.05-point change.

**Fig. 3:**
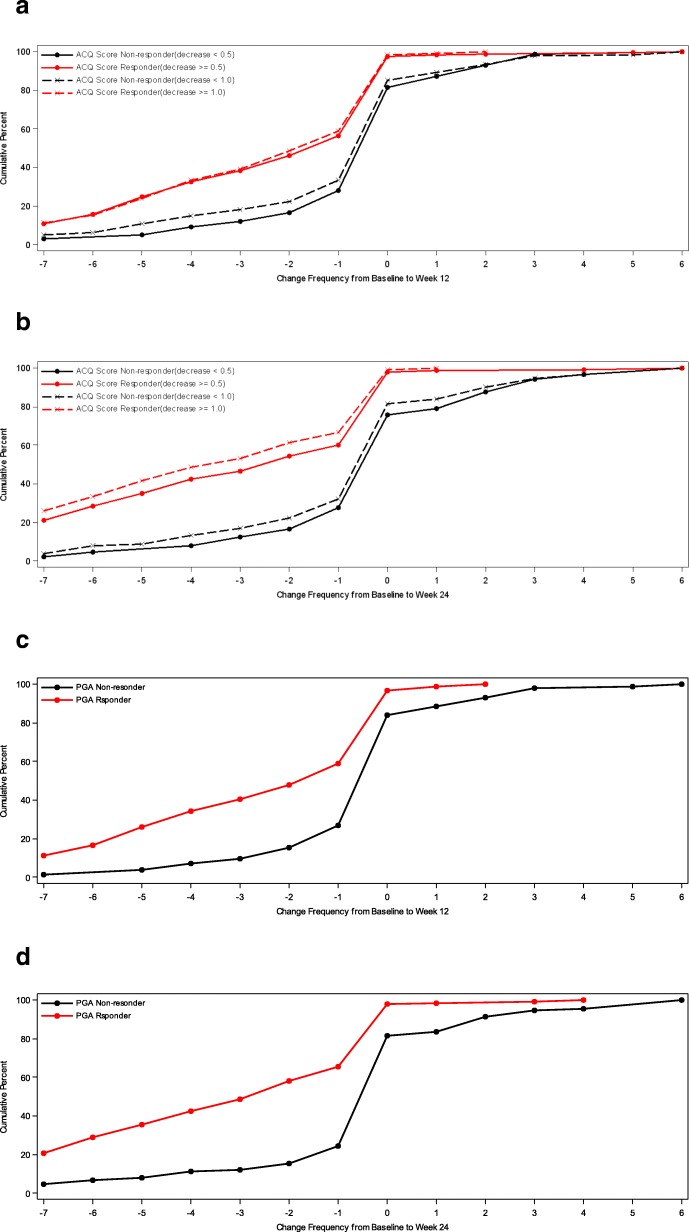
Cumulative Distribution Curves for ASD Symptomatic Days by ACQ Based Responder Criteria and PGA Based Responder Criteria. **a** Baseline to Week 12 by ACQ-5 Response. **b** Baseline to Week 24 by ACQ-5 Response. **c** Baseline to Week 12 by PGA Response. **d** Baseline to Week 24 by PGA Response. PRO analysis set includes all subjects who are enrolled and have Asthma Symptom Diary measurement at baseline and at 12 weeks or 24 weeks

For ASD-based Symptomatic Days, CDF results are summarized for the ACQ-based criteria and for the PGA-based criteria (see Fig. 3). Clearly, there is significant discrimination in Symptomatic Days for most of the distribution of changes for baseline to week 12 and baseline to week 24. Based on the CDF results, the responders demonstrated a decrease of 1.0 to 2.2 Symptomatic Days (over a 7-day period), while non-responders demonstrated only a 0.4 to 0.5- day decrease.

**Fig. 4:**
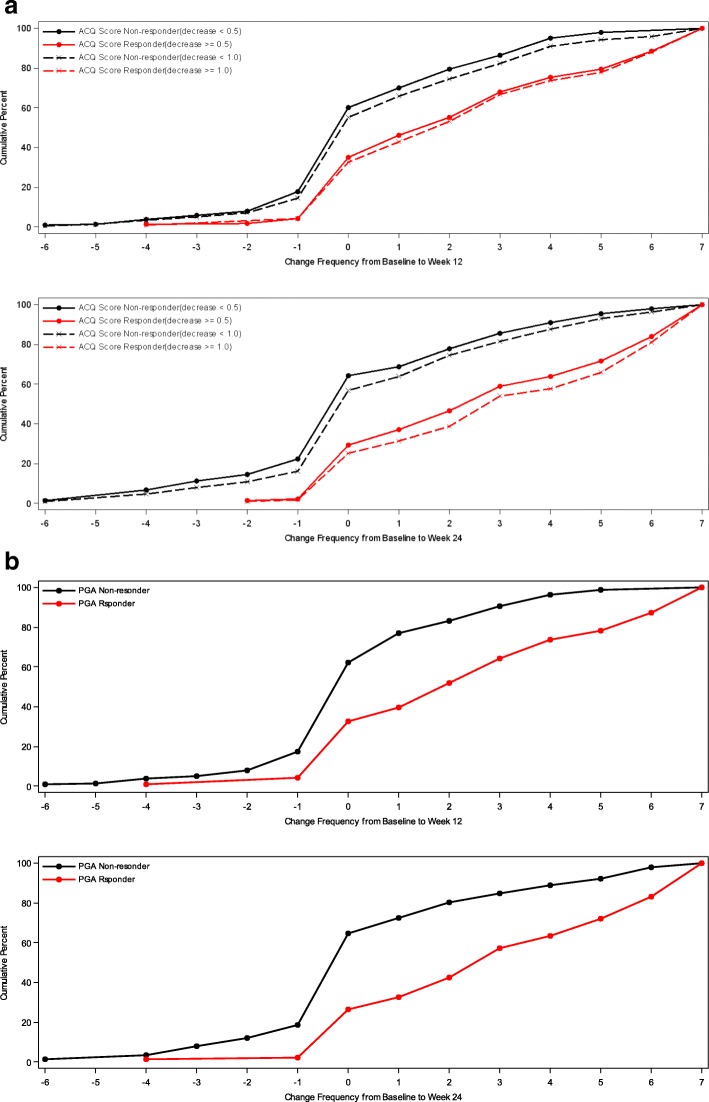
a Cumulative Distribution Curves for ASD Minimal Symptom Days-1 by ACQ Based Responder Criteria PRO analysis set includes all subjects who are enrolled and have Asthma Symptom Diary measurement at baseline **b** Cumulative Distribution Curves for ASD Minimal Symptom Days-1 by PGA Based Responder Criteria. PRO analysis set includes all subjects who are enrolled and have Asthma Symptom Diary measurement at baseline and at 12 weeks or 24 weeks

**Fig. 5:**
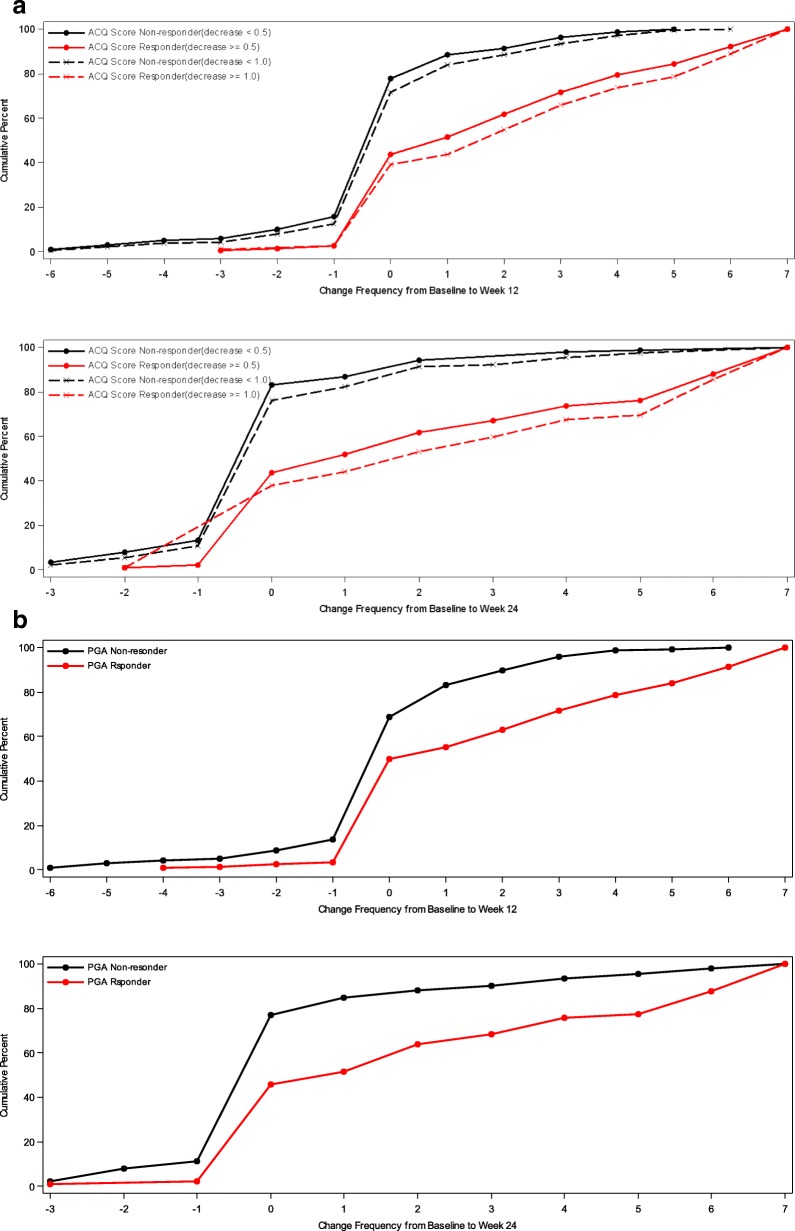
**a** Cumulative Distribution Curves for ASD Minimal Symptom Days-2 by ACQ Based Responder Criteria PRO analysis set includes all subjects who are enrolled and have Asthma Symptom Diary measurement at baseline and at 12 weeks or 24 weeks. **b** Cumulative Distribution Curves for ASD Minimal Symptom Days-2 by PGA Based Responder Criteria. PRO analysis set includes all subjects who are enrolled and have Asthma Symptom Diary measurement at baseline and at 12 weeks or 24 weeks

For ASD-based Minimal Symptom Days, CDF results are summarized for the ACQ-based responder criteria and the PGA-based responder criteria (see Figs. 4 and 5). There is significant discrimination in the distribution of changes from baseline to week 12 and baseline to week 24 Minimal Symptom Days. For Minimal Symptom Days-1 (Figs. 4a and b), based on the CDF findings, the responders demonstrated an increase of approximately 1.0 day (over a 7-day period), while non-responders demonstrated only a 0.2 to 0.5-day decrease. Somewhat larger effects were observed for Minimal Symptom Days-2 (Figs. 5 a and b), with responders demonstrating an increase of 1.7 to 2.6 days (during a 7-day period),while non-responders demonstrated only a 0.2 to 0.4-day decrease.

## Discussion

The ASD was developed to evaluate daytime and nighttime asthma symptom outcomes for clinical trials, and has evidence supporting content validity and good measurement properties [10, 11]. Extensive patient engagement and clinician review and input provided the basis for the development of the ASD. Previous research has demonstrated that the ASD has good evidence supporting reliability and validity, and provides preliminary evidence supporting responsiveness [11]. The current secondary analysis utilizes Phase II clinical trial data to evaluate the sensitivity to change of the ASD, and evaluates several ASD-based responder endpoint definitions. Definitions of days with asthma symptoms and days with minimal asthma symptoms were developed to evaluate treatments for asthma and were evaluated in this study.

These analyses further confirm the responsiveness of the ASD scores. The 7-day average asthma symptom scores demonstrated significant differences between responders and non-responders based on ACQ- and PGA-derived anchors. Consistently, anchor-based measure responders reported changes of between 0.48 to 0.54 points after 12 weeks of treatment, and 0.59 to 0.68 points after 24 weeks of treatment. Non-responders reported changes of only 0.05 to 0.13 points in ASD 7-day average scores after 12 weeks, and 0.06 to 0.15 points after 24 weeks of treatment. The magnitude of these statistically significant effects were large, with effect sizes of 0.80 to 0.90 and 0.98 to 1.13 at 12 and 24 weeks, respectively. These changes in 7-day mean ASD scores were seen regardless of using an ACQ or PGA defined anchor. The current study observed changes in 7-day mean ASD scores are consistent with the changes seen for non-responders (0.0 to − 0.1), but were somewhat larger than responders (− 0.2 to − 0.3) in the Globe et al. [11] study. These differences may be attributable to the absence of an asthma severity inclusion criteria and relatively short follow-up (i.e., 4 weeks) in the Globe et al. [11] study. The intent for the Globe et al. [11] study was to recruit across severity levels consistent with [9] guidelines.

For ASD symptomatic days, responsiveness was demonstrated for both the ACQ defined and PGA defined anchors after 12 and 24 weeks of treatment. The study findings showed decreases in 2.2 to 2.4 symptomatic days after 12 weeks of treatment, and decreases of 2.9 to 3.2 symptomatic days were observed after 24 weeks of treatment for the ACQ and PGA defined anchors. This observed magnitude of change in number of symptomatic days is substantial, and likely reflects important improvements in asthma-related outcomes for patients with moderate to severe asthma. The effect sizes ranged from 0.73 to 1.07. The results suggest that a reduction of 2 to 3 symptomatic days may be important in moderate to severe asthma patients.

As expected, the findings for ASD defined minimal symptom days, also mirrored the effects for ASD based symptomatic days. For example, for ASD Minimal Symptom Days-1, improvements of 2.3 to 2.5 days were seen after 12 weeks of treatment, and 2.9 to 3.2 days after 24 weeks of treatment. The Minimal Symptoms Days-2 version also showed comparable, but slightly attenuated effects at 12 and 24 weeks. Based on these study results, we recommend that minimal symptom days are defined, based on the first version, as the mean of the ASD daily symptoms less than or equal to 1 with no single symptom item score with a response greater than 1. Both definitions of minimal symptom days demonstrated good responsiveness and differentiation across different anchors, however, reductions in symptomatic days is recommended as a clinical trial endpoint as this outcome may resonate more with clinicians and may better reflect outcomes important to patients.

For patient-reported outcomes, guidelines are necessary for interpreting changes in outcomes either for applications in clinical trials comparing treatment and for clinical practice settings. For the FDA and other regulatory agencies, their main concern relates to identifying clinical differences that are greater than minimal and that are important to patients, therefore the responder thresholds were based on PGA score changes of greater than or equal to 1 and ACQ scores of 1.0 or greater. For ASD 7-day average scores, responders may be defined as those patients who report improvements of 0.50 to 0.60 points, based on the results of this study. The patient’s perspective is important for understanding meaningful outcomes, and the two patient-reported anchors used in this study are relevant for identifying responders. Although distribution-based methods may provide insight as to the magnitude of change, they do not provide information as to what level of change is important to patients and clinicians [23]. Clearly, treatment effects in this range of magnitude of change are likely to be important to patients with moderate to severe persistent asthma and clinically important to their clinicians when evaluating treatment effects. The MID for the ASD 7-day average scores were estimated at 0.35, which reflects a 0.58 effect size, and may be considered moderate.

For the ASD defined symptomatic days, decreases of 2 to 3 days are important to patients with moderate to severe asthma. Decreases as small as 1.75 symptomatic days may also be considered meaningful outcomes from a patient’s perspective. Responder definitions based on this magnitude of change are likely to differentiate highly effective from less effective treatments for asthma. Future research is needed to further confirm this responder definition for asthma symptomatic days. Based on the range of asthma-specific anchors included in this study and the CDF analyses, however, these findings are likely to be confirmed with future research.

Similar responder criteria can be derived for different versions of minimal symptom days based on the ASD. Clearly, as expected, minimal symptom days represent the reverse reflection of asthma symptom days. As with the ASD defined symptomatic days, increases of 2 to 3 days with minimal asthma symptoms are likely meaningful for moderate to severe asthma patients and their clinicians. An improvement of two days per week in minimal symptom days may represent an acceptable criterion for defining responders in clinical trials comparing treatments of moderate to severe persistent asthma. However, smaller improvements may also reflect important changes. Future research is needed to further confirm these findings, but given the range of asthma related anchors and various analyses, these results are likely to be confirmed as the results are consistent irrespective of method used.

There are significant challenges in defining responders, based on the magnitude of change considered important by patients, and in evaluating the statistical significance of within-patient changes [13, 19]. Although some researchers apply the MID as the threshold for identifying individual patient responders, higher thresholds of often necessary for defining within-patient responders, given the variability and less reliability in individual patient scores compared to group mean scores. Hays et al. [13] have examined the reliable change index and standard error of measurement for understanding individual patient level changes in health-related quality of life, while Kemmler et al. [19] combine both clinical relevance and statistical significance in evaluating within-patients changes in patient reported outcomes.

Several limitations should be acknowledged related to interpreting the findings of this study. First, the analyses were based on a clinical trial sample, and any demographic or clinical differences of the clinical trial sample and the asthma general population may impact on the generalizability of these results. However, the analysis of sensitivity to change from a clinical trial sample has certain advantages, since there will likely be some patients who improve, remain stable and worsen over the course of the study [23]. Second, the anchors used to evaluate responsiveness and responder definitions are derived from patient reports and may be associated with some measurement bias. Given that the ASD is a PRO measure, the perspective of patients is most important in examining sensitivity to change in clinical status and for understanding the importance of these changes. Finally, multiplicity of statistical tests may be an issue, however, the consistency of findings across different anchors obviates concern associated with multiple statistical tests.

## Conclusion

Interpretation guidelines based on the MID estimates can be used to evaluate clinical significance of mean differences in ASD scores and for determining sample size estimates for clinical trials. The identification of responder definitions and interpretation guidelines provides further insight into individual level treatment benefits. The findings of this study indicate that the ASD is a good symptom assessment tool for asthma clinical trials in patients with moderate to severe persistent asthma. Several responder definitions, based on to the ASD to define symptomatic and minimal symptom days, may be useful in evaluating treatment effects at the individual patient level in clinical trials.

## Abbreviations

ACQ: Asthma Control Questionnaire
ANCOVA: Analysis of covariance
ASD: Asthma Symptom Diary
CDF: Cumulative distribution function
EMA: European Medicines Agency
FDA: Food and Drug Administration
FEV1: Forced expiratory volume in one second
HRQL: Health-related quality of life
MID: Minimal important differences
PGA: Patient Global Assessment
PRO: Patient reported outcome
